# Catalytic, theoretical, and biological investigation of an enzyme mimic model

**DOI:** 10.3906/kim-2104-51

**Published:** 2021-08-27

**Authors:** Gülcihan GÜLSEREN

**Affiliations:** 1 Department of Molecular Biology and Genetics, Faculty of Agriculture and Natural Sciences, Konya Food and Agriculture University Turkey

**Keywords:** Artificial enzyme, peptide, catalytic activity, biomineralization

## Abstract

Artificial catalyst studies were always stayed at the kinetics investigation level, in this work bioactivity of designed catalyst were shown by the induction of biomineralization of the cells, indicating the possible use of enzyme mimics for biological applications. The development of artificial enzymes is a continuous quest for the development of tailored catalysts with improved activity and stability. Understanding the catalytic mechanism is a replaceable step for catalytic studies and artificial enzyme mimics provide an alternative way for catalysis and a better understanding of catalytic pathways at the same time. Here we designed an artificial catalyst model by decorating peptide nanofibers with a covalently conjugated catalytic triad sequence. Owing to the self-assembling nature of the peptide amphiphiles, multiple action units can be presented on the surface for enhanced catalytic performance. The designed catalyst has shown an enzyme-like kinetics profile with a significant substrate affinity. The cooperative action in between catalytic triad amino acids has shown improved catalytic activity in comparison to only the histidine-containing control group. Histidine is an irreplaceable contributor to catalytic action and this is an additional reason for control group selection. This new method based on the self-assembly of covalently conjugated action units offers a new platform for enzyme investigations and their further applications. Artificial catalyst studies always stayed at the kinetics investigation level, in this work bioactivity of the designed catalyst was shown by the induction of biomineralization of the cells, indicating the possible use of enzyme mimics for biological applications.

## 1. Introduction

Our increasing demand for natural like biomaterial has resulted in a growing interest in the development of artificial catalysts [1]. These special macromolecules can be used repeatedly to facilitate well-optimized catalytic function [2]. Therefore, enzyme mimicry studies attracted remarkable interest for designing synthetic catalysts with improved stability and function by exploiting the modifiable features of the synthetic materials [3]. However, we are still far from attaining the catalytic success of the native enzymes due to their well-optimized mechanism. Hence, there is a significant requirement for the development of a new type of catalysts for understanding the elaborate nature of these catalytic proteins. 

So far, various macromolecules were employed as artificial catalysts [4]. Natural-like biomaterials are always considered as an advantageous toolbox for a wide variety of applications [5]. Most common examples of enzyme mimics were designed by using organic macromolecules, antibodies, polymers, and nanoparticles [. Peptide nanomaterials hold an important place among these artificial scaffolds due to their structural similarity with the native proteins [10], well-optimized synthetic protocols, and modifiable structure [11]. They can be converted into multifunctional nanomaterials-nanofibers or micelles- simply by the attachment of a hydrophobic tail [12]. In this way, a bioactive group carrying nanoparticles assemble into nanofibers, and nanostructure formation leads to improved bioactivity via nanosurface phenomena [13]. Hydrophobic tails are usually selected from known fatty acids, which are also practical for membrane transportation as well. Bioactive nanogroups on peptide nanofibers can be tailored by considering the desired function. Small active units can also be inserted into these nanofibers, which means that these molecules can be applied simultaneously for multiple functions. In addition to the biological function, fiber formation can be induced with various triggers such as pH, counter ion, counter sequence, and light [14]. These features make them action site-specific as well. Depending on the pH or active biomolecule content of the targeted tissue, peptide-based agents can be manipulated for specific action.

The catalytic triad is a most common sequence found in various enzyme types (i.e. hydrolases and esterases) [15]­ü and this set of coordinated amino acids, histidine/serine/aspartic acid (DHS), is frequently used in catalytic sites of artificial mimics [16]. These models were used to recapitulate the function of the catalytic triad containing enzymes [17]. The proposed structure in our new design will form the basis for nanofiber-based catalytic activity as well as the function. To achieve this goal DHS sequence was linked to the hydrophobic tail for nanofiber formation and the presentation of covalently linked active groups on nanofiber surfaces were investigated in terms of catalytic activity. The catalytic and biologic function of the designed peptide amphiphile was tested with the new design and the molecular activity of the model sequence was explained by the molecular calculations as well. In this context, following the kinetic demonstration of its biocatalytic activity, the designed artificial mimic was investigated with molecular modelling calculations to illustrate the contribution of each active site. At present, artificial catalysis studies are only at the catalytic activity phase and the biological activities of these artificial catalysts were not tested in model cell applications. Our goal in this work is to develop a biomimetic enzyme structure that can be applied to living cells, mimicking not only the chemical function but also the biological functions of the enzyme. Thus, a developed artificial model can be applied as a potential alternative to natural enzymes for therapeutic applications.

## 2. Materials and methods

9-Fluorenylmethoxycarbonyl (Fmoc) protected amino acids, lauric acid, [4-[a-(20,40-dimethoxyphenyl) Fmoc-amino methyl] phenoxy] acetomidonorleucyl-MBHA resin (Rink amide MBHA resin), 2-(1H-Benzotriazol-1-yl)-1,1,3,3 tetramethyluronium hexafluoro- phosphate (HBTU), diisopropylethylamine (DIEA), and p-nitrophenyl acetate (PNPA) were purchased from Merck and ABCR. Plate reader plates (96-well) were purchased from BD. All other chemicals and materials used in this study were analytical grade and obtained from Invitrogen, Fisher, Merck, Alfa Aesar, and Sigma Aldrich.

### 2.1. Synthesis and characterization of peptide amphiphiles (PA)

Functionalized peptide molecules were synthesized manually by standard solid-phase Fmoc peptide synthesis chemistry. The catalytic triad mimetic peptide and lauryl-VVAGDHS peptide was constructed on MBHA Rink Amide resin at 0.25 mmol scale. Amino acid couplings were done with 2 equivalents of Fmoc-protected amino acid (or lauric acid), 1.95 equivalent of HBTU, and 3 equivalent of DIEA for 2 h. Fmoc removal was performed with 20% piperidine/dimethylformamide (DMF) solution for 20 min. Cleavage of the peptides from the resin was carried out with a mixture of TFA: TIS: H_2_O in the ratio of 95:2.5:2.5 for 3 h. Excess TFA was removed by rotary evaporation. The remaining viscous peptide solution was triturated with ice-cold ether and the resulting white product was freeze-dried. Peptides were characterized by Agilent 6530 quadrupole time of flight (QTOF) mass spectrometry with electrospray ionization (ESI) source equipped with reverse-phase analytical high performance liquid chromatography (HPLC) with Zorbax Extend-C18 2.1 50 mm column for basic conditions and Zorbax SB-C8 4.6 100 mm column for acidic conditions. An optimized gradient of 0.1% formic acid/water and 0.1% formic acid/acetonitrile for acidic conditions and 0.1% ammonium hydroxide/water and 0.1% ammonium hydroxide/acetonitrile for basic conditions were used as mobile phase for analytical HPLC, respectively. The peptide was synthesized with 97% purity level. 

### 2.2. Self-assembled nanofiber formation and enzyme kinetics 

Nanofibers were formed by the elevation of the pH to 7.4. To perform kinetic experiments 2.5 x 10 **^–^** ^5 ^M peptide was dissolved in 10 mM HEPES at pH 7.4 and incubated for fiber formation. The stock solution of the pNPA (p-nitrophenylacetate) were prepared in DMSO and diluted to 10 different concentrations between 1 x 10 **^–^** ^4^ M to 5 x 10 **^–^** ^3^ M and mixed with enzyme mimetic peptides, respectively. Biotech Epoch 2 plate readers were utilized for activity measurements. Immediately following mixing, the enzymatic reaction rate is obtained by spectral measurement of PNP (paranitrophenyl- which is the resulting product after enzymatic reaction) at 410 nm. Reaction rates were calculated for each concentration with an extinction coefficient of 10.166 M^–1^cm^–1^ and fitted into the Michaelis-Menten equation (v_0 _= kcat[E]_0_[S]_0_/(K_M_ + [S_0_])). Graphpad 5 was used for graphical fitting and calculations. 

### 2.3. Microscopy imaging

Transmission electron microscopy (TEM) was employed to visualize nanofibers and the resulting network structure. TEM samples were prepared on the cleaned cover glass surface by elevating the pH of the peptide solution. TEM images were acquired with FEI Tecnai G2 F30 TEM at 300 kV. Samples for TEM were prepared by mixing on a 200-mesh carbon TEM grid for 3 min followed by 2 wt % uranyl-acetate staining for 1 min and drying immediately under nitrogen gas. 

### 2.4. Circular dichroism 

To investigate the secondary structure of peptide nanofibers, circular dichroism (CD) spectra of 3 x 10^–5^ M peptide amphiphile, were measured at room temperature from 300 nm to 190 nm with 0.1 nm data interval and 500 nm/min scanning speed. The results were converted to and represented as the mean residue ellipticity.

### 2.5. Theoretical calculations 

The DHS sequence were theoretically studied by a semi-empirical molecular orbital method (called parameterized model-3 (PM3) [18] with the restricted Hartree−Fock (RHF) [19] formulation. After geometrical minimization, single point energy (SPE) calculation using density functional theory (DFT) [19] with B3LYP (Becke, three-parameter, [20] Lee−Yang−Parr [21,22]) exchange-correlation potential was performed at the basis set 6- 31G^+^(d) [23]. These calculations yielded the frontier molecular orbitals (highest occupied molecular orbital (HOMO) and lowest unoccupied molecular orbital (LUMO)) and electrostatic potentials. The electrostatic potential maps of the active sequence were calculated to determine their chemical activity and electrostatic interactions with other molecules. The program packages Gaussian 09 [24] and GaussView5 [25] were used for theoretical calculations and input and visualization, respectively.

### 2.6. Bioactivity study

Biomineralization and calcium phosphate tests with SaOS-2 cells were carried out on 1 mM DHS-PA 24 well-plates. Peptide was gelated by using DMEM medium (pH 7.4). DHS-PA nanofibers were formed on culture plate surfaces as described above and coated surfaces were first incubated at 37 °C. Coatings were then allowed to dry in a laminar hood overnight. For bioactivity test, SaOS-2 cells were seeded on 24-well plates at a density of 4 × 10^4^ cells/cm^2^ in DMEM with 10% FBS. Calcium deposition on the surfaces was measured on day 7 using Alizarin Red as quantitative colorimetric staining[26]. Briefly, cells were fixed with ice-cold ethanol for 1 h and stained with 40 mM Alizarin-Red S for 15 min. After washing 4−5 times with double-distilled water to remove nonspecific Alizarin-Red binding, cells were imaged with Zeiss optical microscope. 

## 3. Results and discussion 

**Figure 1:**
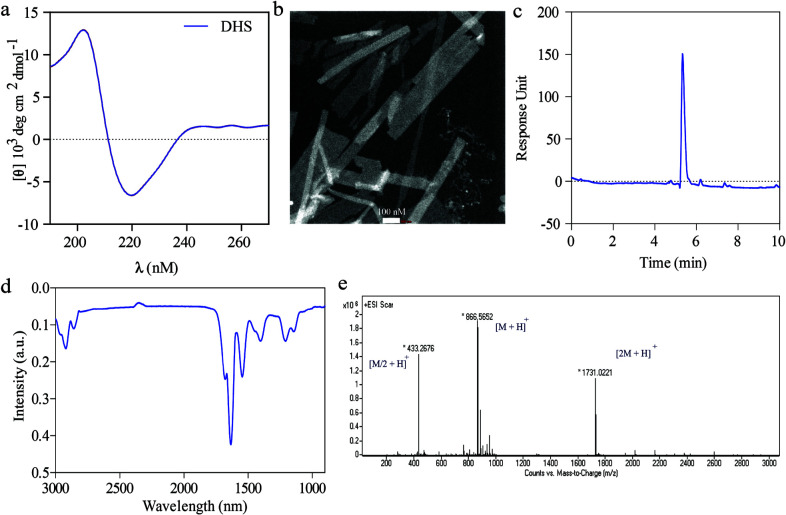
Chemical and the structural characterization of the peptide amphiphile molecule a) Circular dichroism graph, b) TEM image of the nanofibers c) LC graph of peptide solution. HPLC chromatogram of peptide. Absorbance at 220 nm vs. retention time graph. d) FTIR analysis of peptide DHS nanofibers e) Mass spectrometry of peptide after subtracting mass spectra of water sample at that time interval. [M + H]^+^ (calculated) = 866.49 [M + H]^+^ (observed) = 866.5652, [2M + H]^+^ (calculated) = 1731.98 [2M+H]^+^ (observed) = 1731.0221, [M/2 + H]^+^ (calculated) = 433.745 [M/2 + H]^+^ (observed) = 433.2676 .

The solid phase peptide synthesis technique was used to prepare enzyme mimetic DHS-PA (C_12_-VVAGDHS-Am) and the purity of the obtained product were characterized by LC-MS chromatography (Figure 1). Mass spectrometry of results were obtained and calculated as follows; [M + H]^+^ (calculated) = 866.49 [M + H]^+^ (observed) = 866.5652, [2M + H]^+^ (calculated) = 1731.98 [M + H]^+^ (observed) = 1731.0221, [M/2 + H]^+^ (calculated) = 433.745 [M/2 + H]^+^ (observed) = 433.2676 . The nanofiber formation of DHS-PA was evaluated by conducting spectral and microscopic analysis. The secondary structure formation of peptide amphiphiles was evaluated by circular dichroism analysis, beta-sheet specific peaks were obtained around 205 and 220 nm indicating the hydrogen bond formation among the peptide monomers (Figure 1). TEM imaging revealed the sheet-like nanofiber morphology of the designed peptides and the width of the sheet-like nanostructures was about 100–200 nm. This morphology can be seen in short peptides and their spatial organization in the aqueous environment leads the lateral organization rather than cylindrical fibrous structure [12]. The secondary structure formation was indicated via Fourier transform infrared (FTIR) spectroscopy as another identification technique, the spectra obtained from freeze dried peptide nanofibers showed an amide peak at around 1630 cm^−1^ accompanied by a broad band at 1680 cm^–1^, indicating sheet-like secondary structure formation of nanostructures [27].

Enzyme kinetic evaluation of the peptide amphiphile based artificial enzyme was studied with Michaelis−Menten kinetics. The catalytic action is generated by coordinative activity among the amino acids. In the native catalytic triad, the acidic side chain of aspartic acid polarizes the imidazole side chain of the histidine base to activate the hydroxyl group of the serine as a nucleophile [28]. This combinatory action reduces the pKa of the nucleophilic radical group of the serine which then facilitates the cleavage. 

**Figure 2:**
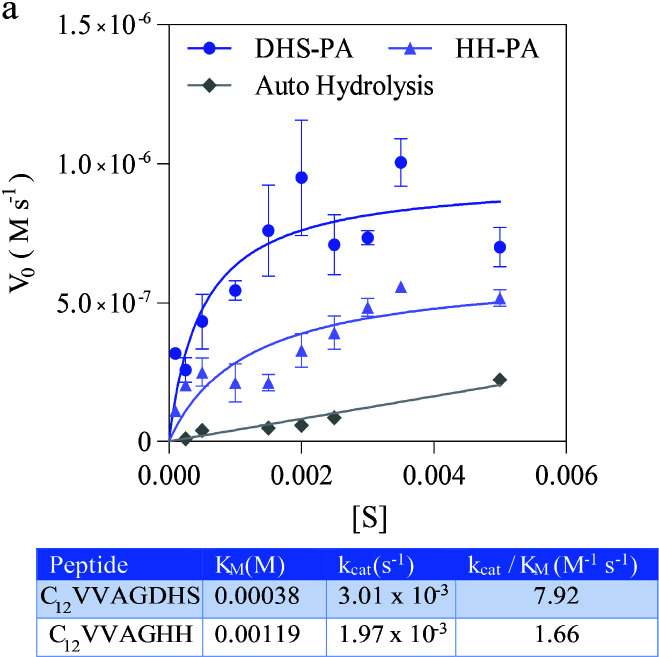
Michaelis−Menten graph and the kinetics table of enzyme kinetics studies of enzyme mimics.

The saturation profile of the enzyme mimic was illustrated by spectroscopic measurement of catalysis of pNPA versus time. Ten different concentrations were evaluated during this measurement and it has been shown that after administration of substrate, catalysis reaches the saturation profile indicating enzyme-like kinetics behaviour of the designed enzyme mimic. The catalytic turnover constant (k_cat_) of the substrate was calculated as 3.01 x 10^−3 ^s^−1^ and the binding constant (K_M_) have found 0.38 mM (Figure 2). The efficiency of cooperative activity among the active amino acids was investigated by a control group (C_12_VVAGHH). The control group was decorated with two histidine moieties to test whether the catalytic action is coming from cooperative interaction of DHS rather than general base characteristics of histidine. To create a similar environment, two histidine moieties were administered for cooperativity. The catalytic efficiency was found ~4 times better than the control group, DHS-PA provided better catalytic turnover and substrate coordination microenvironment for the model substrate. It has shown that cooperative action is responsible for an enzyme-like kinetic profile but the existence of three action units is critical to generate a better catalytic profile *.*

Compared to literature, a moderate catalytic activity can be generated by the peptide-based enzyme mimic decorated by covalently conjugated amino acids (Table). Previous studies have shown that hydrophobic backbone support is another critical parameter for catalytic efficiency, insertion of the hydrophobic amino acids in between action units resulted in significant improvement in catalytic efficiency [34]. In this study, minimal domain of action was investigated to understand its potential in the absence of external support. In comparison with reported enzyme mimics, this new design has shown similar rates at neutral pHs (Table). Interestingly, the calculated binding constant of the enzyme mimic has found very low, which can be interpreted as that covalently linked action units enable optimum environment for substrate binding. 

**Table:** Reported peptide-based enzyme mimics.

Peptide enzyme mimic	kcat / KM (M–1 s–1)	Reaction pH	References
DHS-PA	7.92	7.4	This study
Q11HR-NH2	0.15	7.0	[33]
Ac-IHIHIYI-NH2	355.00	8.0	[34]
Ac-IHIHQYI-NH2	15.76	7.3	[35], [36]
HKH-LLLAAA(K)-C16	19.76	7.3	[37]

The enzyme-like activity of the covalently conjugated DHS and control group were tried to be understood by theoretical calculation. The initial energy minimization calculation of DHS modules was done by a semi empirical molecular orbital method PM3 with the Restricted Hartree−Fock formulation. This semi empirical calculation was followed by geometry optimization calculation using density functional theory with B3LYP exchange-correlation potential was performed at the basis set 6-31G^+^(d). 

**Figure 3:**
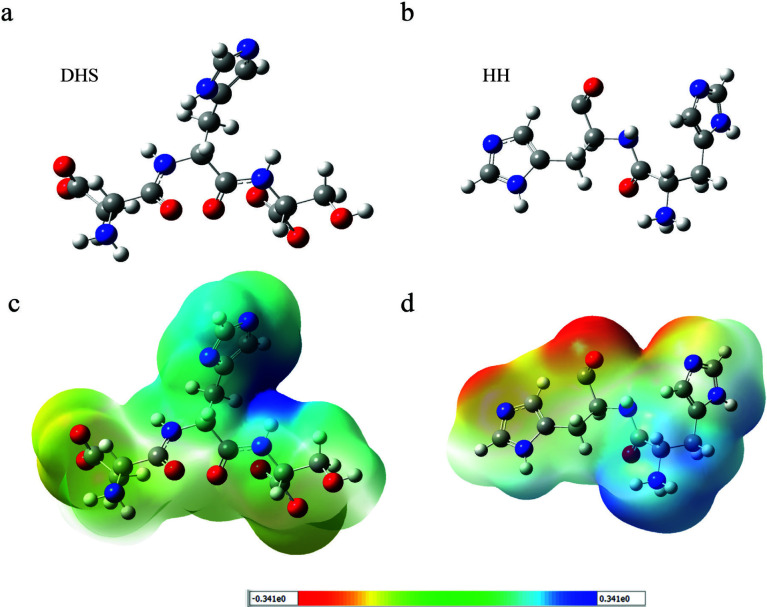
Optimized molecular geometry of the a) active peptide group b) control group peptide sequence. Surface electron density plot of the active site of c) DHS and d) noncovalent DHS at the DFT\B3LYP\6-31G+ level of calculation.

The most probable structural organization of were obtained after geometry optimization calculations, these results indicated that the Nδ side of the imidazole group was tilted towards Asp and Nε were located closer to Ser (Figure 3). The optimized geometry of the peptide sequence has shown that interacting groups of catalytic triad are located closer for optimal activity. The orientation and placement of action units in the defined space of an active pocket is a hallmark of enzymes and of catalytic triad activation. Similarly, histidine modified control unit were calculated to make a comparison in between designed active site and only histidine unit. 

**Figure 4:**
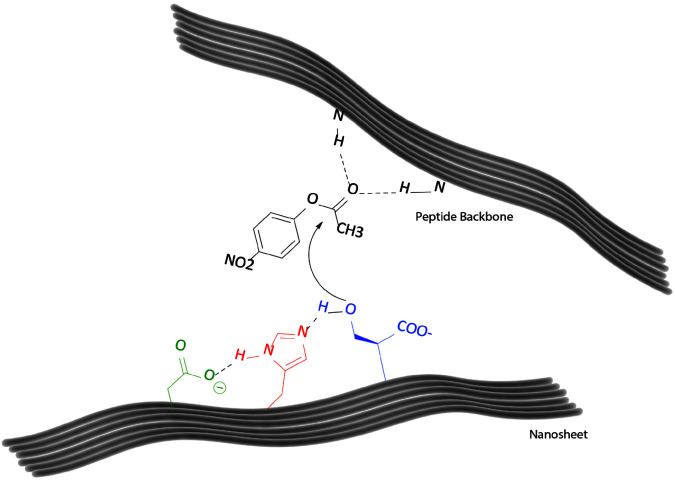
Proposed mechanism of action of DHS-PAs.

The synergistic catalysis is activated by the interaction of the different biomolecular groups. This simultaneous action leads a dramatic decrease in the HOMO-LUMO gap that causes significant acceleration of the catalysis reaction [29,30]. This approach was evaluated for various types of artificial catalysis studies [31,32]. The theoretical calculations yielded the frontier molecular orbitals, and these results were used for investigation of the catalytic activity-molecular structure relation. The ∆E value of the HOMO-LUMO gap was calculated as 6.25 eV. For the control group ∆E value was calculated as 15.51 eV. The closer HOMO−LUMO regions contribute to the reactivity of the molecule because of the decrease in the energy required for the reaction. The obtained decrease in the HOMO-LUMO gap was interpreted as the reason for the enzyme-like catalytic activity of the enzyme mimics. These results indicated that, the imidazole moieties are mainly responsible for the polarization of the serine and therefore nucleophilic activity of the set of coordinated amino acids. According to electrostatic potential (ESP) map, the electron density was located around mostly Asp residue, the blue color nearby the His indicated positive charge around this unit. This result can be interpreted as activation of the His to abstract proton from Ser for nucleophilic action (Figure 3). According to ESP mappings of control group, the imidazole units did not displayed that level of positivity, the lower activity can be explained with the absence of charge-relay network among the action units (Figure 4).

**Figure 5:**
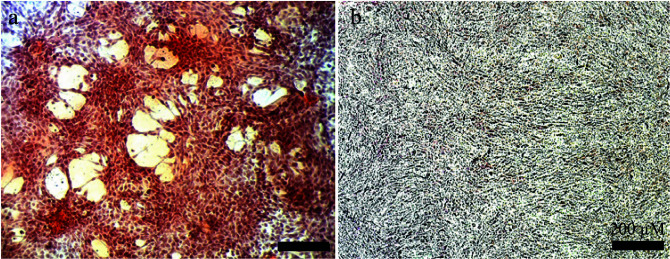
Bioactivity of peptide-based enzyme mimic. Alizarin red staining results of the a) DHS treated peptide amphiphile, b) the nontreated control group.

The biological activity of the enzyme mimic was evaluated with phosphatase-like activity. Most of the serine proteases employ a catalytic triad to facilitate catalytic action. In this part of the study, the designed enzyme mimic was used to cleave phosphodiester bonds for the generation of inorganic phosphates. The resulting inorganic phosphates are deposited on the cell surface for CaP biomineral formations. This process is the key regulator of osteogenesis. The cells treated with DHS peptide have resulted in calcium phosphate mineral deposition like native phosphatase. Besides catalytic activity, the enzyme mimicking peptide catalysts have shown bioactivity by inducing biomineralization and supporting viability (Figure 5). The mineral deposition was evidenced by alizarin red staining, calcium deposited SaOS-2 cells were observed as red staining. 

## 4. Conclusion 

An artificial catalyst model was developed by the decoration of peptide amphiphiles with covalently conjugated action units of the catalytic triad. The enzyme-like kinetic profile of the peptide mimetic design was illustrated via the calculation of Michaelis–Menten graphs and remarkable rates were observed in catalytic turnover and binding constants. It was shown that the success of the catalytic activity is strongly dependent on the peptide sequence and the cooperative activity among these amino acids. Hence, the obtained catalytic efficiency can be improved by simple sequence alterations or by administration of supporting units. Kinetics study was followed by theoretical calculations, optimized molecular structure for the catalytic action, and remarkable reduction in the HOMO-LUMO gap was shown with the computational method. The bioactivity of the enzyme-like was also illustrated by testing its phosphatase-like activity and this peptide resulted in biomineral deposition on the cells. This versatile approach will provide a promising platform for understanding elaborate catalytic mechanisms of the enzymes and for developing better artificial substitutes in the future.
